# Characterization and engineering of plastic-degrading polyesterases jmPE13 and jmPE14 from *Pseudomonas* bacterium

**DOI:** 10.3389/fbioe.2024.1349010

**Published:** 2024-02-14

**Authors:** Xiaoli Zhou, Xianmin Zhou, Zhiqiang Xu, Mingxia Zhang, Honghui Zhu

**Affiliations:** Key Laboratory of Agricultural Microbiomics and Precision Application (MARA), Key Laboratory of Agricultural Microbiome (MARA), Guangdong Provincial Key Laboratory of Microbial Culture Collection and Application, State Key Laboratory of Applied Microbiology Southern China, Institute of Microbiology, Guangdong Academy of Sciences, Guangzhou, China

**Keywords:** polyester, hydrolase, enzyme engineering, PET, PBAT

## Abstract

Polyester plastics are widely used in daily life, but also cause a large amount of waste. Degradation by microbial enzymes is the most promising way for the biobased upcycling of the wastes. However, there is still a shortage of high-performance enzymes, and more efficient polyester hydrolases need to be developed. Here we identified two polyester hydrolases, jmPE13 and jmPE14, from a previously isolated strain *Pseudomonas* sp. JM16B3. The proteins were recombinantly expressed and purified in *E. coli*, and their enzymatic properties were characterized. JmPE13 and jmPE14 showed hydrolytic activity towards polyethylene terephthalate (PET) and Poly (butylene adipate-co-terephthalate) (PBAT) at medium temperatures. The enzyme activity and stability of jmPE13 were further improved to 3- and 1.5-fold, respectively, by rational design. The results of our research can be helpful for further engineering of more efficient polyester plastic hydrolases and their industrial applications.

## 1 Introduction

Polyester plastics are widely used in our daily life due to their good properties such as durability, waterproofing, and transparency. At the same time, their wastes have also caused serious environmental pollution (MacLeod et al., 2021). Polyethylene terephthalate (PET) consists of ethylene glycol (EG) and terephthalate (TPA) and is the most popular plastic used in the packaging of beverages and food, as well as medical devices and textiles. Poly(butylene adipate-co-terephthalate) (PBAT) is composed of TPA, adipic acid, and 1, 4-butanediol, and is widely used in agricultural mulch films and plastic bags. Although these polyesters are considered biodegradable, the natural degradation rate of them can be very slow (Jimenez et al., 2022). Microorganisms and their secreted enzymes can degrade and transform plastic waste, making bio-upcycling the most promising approach for waste plastic treatment. To bio-upcycle plastics efficiently, it is still necessary to discover the microorganisms and enzymes that are capable of degrading plastics and modify them to improve their catalytic efficiency (Ali et al., 2021; Tamoor et al., 2021).

Although the appearance of plastics is not long ago, some enzymes with promiscuous activity derived from soil or marine microorganisms as well as metagenomes have been found to hydrolyze the ester bonds of plastics such as PET (Zhu et al., 2022; Qiu et al., 2023; Tournier et al., 2023). The enzymes with broad substrate spectrum, including lipases, esterases, and cutinases, have been reported to depolymerize PET mainly producing mono-2-hydroxyethyl terephthalic acids (MHET). Among them, cutinases generally have good thermostability and catalyze PET degradation at a temperature of about 50°C (Sui et al., 2023). In 2016, IsPETase and IsMHETase from *Ideonella sakaiensis* 201-F6 were found to act synergistically to hydrolyze PET to TPA under mesophilic conditions (Yoshida et al., 2016; Palm et al., 2019). PBAT shares some structural similarities with PET, and some enzymes have both PET and PBAT hydrolytic activities such as Ples from the marine microbial consortium I1 (Li et al., 2022; Meyer Cifuentes et al., 2022) and TfCut from *Thermobifida fusca* (Chen et al., 2008; Roth et al., 2014; Yang et al., 2023). Some other enzymes have only been reported to have PBAT hydrolytic activity, such as PpEst from *Pseudomonas pseudoalcaligenes* was reported to degrade PBAT to terephthalatebutanediol monoester (BT) (Wallace et al., 2017). The discovery of these enzyme activities provides a material and theoretical basis for biocatalysis of plastic degradation.

Due to the unsatisfactory activity and stability of native enzymes, enzyme engineering efforts have been made to modify these hydrolases to improve their catalytic properties (Samak et al., 2020; Lu et al., 2022; Wei et al., 2022; Blazquez-Sanchez et al., 2023; Li et al., 2023; Liu et al., 2023; Shi et al., 2023). For example, the mutant LCC^ICCG^ obtained by combinatorial site-directed mutagenesis of LCC, a leaf-branch compost metagenome-derived cutinase, can depolymerize PET by 90% in 10 h at 72°C (Tournier et al., 2020). DuraPETase is a mutant of IsPETase that has been engineered by the computational redesign strategy (GRAPE) and has increased its activity by 300 times at mild temperatures (Cui et al., 2021). Yang et al. engineered TfCut through a double mutation strategy to render a more flexible substrate-binding pocket, enabling it to completely hydrolyze PBAT into TPA within 48 h (Yang et al., 2023). In addition, techniques such as cell surface display and nano-immobilization have also been used to improve the catalytic performance of PETases (Jia et al., 2021; Jia et al., 2022). Great progress has been made in the research of plastic-degrading enzymes, but it is still not enough to meet the needs of industrial applications. Discovering more polyester hydrolases and improving their properties will further contribute to polyester plastic upcycling (Xu et al., 2023).

In this study, we identified two potential polyester hydrolases, jmPE13 and jmPE14, from a previously isolated strain *Pseudomonas* sp. JM16B3. The proteins were recombinantly expressed and purified in *E. coli*, and their enzymatic properties were characterized. JmPE13 and jmPE14 showed hydrolytic activity toward PET and PBAT at medium temperatures. The enzyme activity and stability of jmPE13 were further improved by rational design.

## 2 Materials and methods

### 2.1 Materials

Polyethylene terephthalate (PET) and polybutylene adipate co-terephthalate (PBAT) were purchased from Macklin (Shanghai, China). Bis (2-hydroxyethyl) terephthalate (BHET) was purchased from Aladdin (Shanghai, China). Terephthalatebutanediol monoester (BT) was purchased from Aikon Biopharmaceutical R&D Co. Ltd (Jiangsu, China). Mono-(2-hydroxyethyl) terephthalic acid (MHET) was synthesized according to the protocol by Palm *et al.* (Palm et al., 2019). PET and PBAT semicrystalline films and microplastics samples were prepared according to the previously reported methods (Cui et al., 2021).

### 2.2 Cloning, expression, and purification of enzymes

The full-length gene of jmPE13 and jmPE14 without the signal peptide sequence was codon-optimized (Supplementary Table S1), synthesized, and cloned into the pET-32b expression vector, by GENEWIZ (Suzhou, China). The mutants were generated by using the site-directed mutagenesis on wild-type jmPE13. The site-directed mutation of jmPE13 was carried out by whole-plasmid PCR using the primers listed in Table 1. The PCR products were digested with DpnI to remove the parent plasmid and purified with a PCR purification kit. The construct was transformed into *E. coli* BL21 (DE3) to express the protein. LB liquid medium containing ampicillin (100 μg/mL) was inoculated with a starter culture. Cultures were grown at 37°C until the OD_600_ was approximately 0.6. Isopropyl-beta-D-thiogalactopyranoside (IPTG, 0.1 mM final concentration) was added to induce protein expression. Then they were incubated overnight at 16°C with shaking at 200 rpm. Cells were harvested by centrifugation for 10 min at 8,000 g at 4°C, and then suspended in 50 mM Tris-HCl (pH 8.0). After sonication, the cell suspension was centrifuged (4°C) at 12,000 g for 20 min, and the supernatant was subjected to nickel-chelating chromatography.

**TABLE 1:** Primers for constructing the mutants.

Mutant	Primers	Template
M1	5′-AGCAACAGCAGCACGAGCGCCCTGAGGAACAAAATTGATAGCACCCGC-3′ 5′-AATTTTGTTCCTCAGGGCGCTCGTGCTGCTGTTGCTCTGACTAATCAG-3′	jmPE13
M2	5′-GATTATCTGATTAGTCAGAGCACGAGCGCCCTGAGGAAC-3′ 5′-CCTCAGGGCGCTCGTGCTCTGACTAATCAGATAATCCAG-3′	M1
M3	5′-ATT​AGT​CAG​TGC​AAC​AGC​CGC​ACG​AGC​CCG​CTG​TAT​AAC​AAA​TGC​GAT-3′ 5′-GGT​GCT​ATC​GCA​TTT​GTT​ATA​CAG​CGG​GCT​CGT​GCG​GCT​GTT​GCA​CTG-3′	jmPE13
M4	5′-AGCAACAGCAGCACGAGCCCGCTGTATAACAAAATTGATAGCACCCGC-3′ 5′-AATTTTGTTATACAGCGGGCTCGTGCTGCTGTTGCTCTGACTAATCAG-3′	jmPE13

### 2.3 Enzyme activity assays

When using *p*-nitrophenol caprylate (*p*NP-C8) as substrate, the reaction system contained 50 mM Tris-HCl buffer at pH 8.0, 10 mM *p*NP-C8, and 0.2 mg/mL enzyme protein. The release of *p*-nitrophenol was recorded by measuring the absorbance at 405 nm in a Multiskan GO microplate reader at 37°C. When using PET or PBAT semicrystalline microplastics as substrate, the reaction system contained 50 mM Tris-HCl buffer at pH 8.0, 1 mg/mL microplastics, and 0.2 mg/mL enzyme protein. The mixture was incubated at 30°C with shaking at 1,000 rpm and the products were analyzed by high-performance liquid chromatography (HPLC). All experiments were performed three times and corrected for the subtraction of substrate self-decomposition, that is, buffer without enzyme protein as a control.

### 2.4 The effect of pH and temperature on enzyme activity

The effect of pH and temperature on the activity of the enzyme (0.2 mg/mL) was tested using *p*NP-C8 (10 mM) as substrate. The effect of pH on enzyme activity was determined by measuring the activity at a pH ranging from 4.5 to 9.2. 50 mM citrate-sodium citrate buffer was used for pH 4.0-6.6, 50 mM phosphate buffer was used for pH 6.6-7.8, and 50 mM Tris-HCl buffer was used for pH 7.8-9.2. The effect of temperature on enzyme activity was examined across the range of 25°C–65°C, in 50 mM Tris-HCl buffer at pH 8.0.

### 2.5 Thermal stability

Thermal inactivation of the enzyme proteins was examined at 40°C. The purified proteins (1 mg/mL in 50 mM Tris-HCl buffer at pH 8.0) were incubated at 40°C and sampled at different intervals and then cooled on ice for 10min. Their residual enzyme activities were assayed at 37°C using *p*NP-C8 as substrate as described above.

### 2.6 Enzyme kinetic assays

Kinetic parameters of the enzymes for BHET were determined in 50 mM Tris-HCl buffer at pH 8.0 containing 0.03–2.4 mM of BHET and 0.2 mg/mL enzyme protein. The mixture was incubated at 37°C for 2 h and the products were analyzed by HPLC. The kinetic constants were obtained through nonlinear regression based on the Michaelis-Menten equation.

### 2.7 HPLC

HPLC was used to analyze the products according to the methods described previously (Tournier et al., 2020) with some modifications. Briefly, 150 μL of the sample was mixed with 150 μL of methanol and 6.5 μL of 6 N HCl and filtered through a 0.22 μm filter before running HPLC. Measurement of the products was performed using an Agilent 1,260 Infinity II equipped with an InertSustain C18 column (4.6 × 250 mm, 5 μm) with a detection wavelength of 240 nm. The column oven was held at 25°C. The mobile phase was 1 mM H_2_SO_4_ in water with a gradient of methanol (30%–90%) at 1 mL·min^−1^.

### 2.8 Scanning electron microscope (SEM) analysis

The post-consumer PET bottles were cut into 6 mm slices and incubated with 0.2 mg/mL of enzyme protein or protein-free 50 mM Tris-HCl buffer as treated and control groups, respectively. After incubating at 30°C for 7 days, the slices were dried in air and coated using gold. The surfaces of the slices were observed under SEM (Hitachi S-3400N) at different magnifications.

### 2.9 Sequence alignment and phylogenetic analysis

The protein sequences of the characterized PET-hydrolases were obtained from the UniProt database (https://www.uniprot.org/uniprotkb/). The amino acid sequences were conducted with multiple sequence alignment using the Clustal Omega web server (https://www.ebi.ac.uk/Tools/msa/clustalo/) (Sievers and Higgins, 2018). The results were rendered by ESPript 3.0 (Gouet et al., 1999). The neighbor-joining phylogenetic tree was created by MEGA-X (Kumar et al., 2018), and the figure was generated by the iTOL web server (https://itol.embl.de/) (Letunic and Bork, 2007).

### 2.10 Homology modeling, molecular docking, and molecular dynamic simulations

The homology model structures of jmPE13 and jmPE14 were created by the SWISS-MODEL web server (https://swissmodel.expasy.org/) (Waterhouse et al., 2018) using the crystal structure of PET2 mutant (PDB entry: 7ECB) (Nakamura et al., 2021) as the template. Pymol software (The PyMOL Molecular Graphics System, Version 1.8 Schrödinger, LLC, De Lano Scientific, San Carlos, CA, United States of America) was used to view the structure and generate figures.

AutoDock 4.1 (The Scripps Research Institute, La Jolla, CA, United States) (Morris et al., 2009) was used to predict the binding modes of jmPE13 and jmPE14 with BHET. AutoDockTools 1.5.6 was used to prepare the proteins and ligands for docking procedure. Kollman charges and polar hydrogens were added. AutoGrid was used to generate the grid maps. The grid dimensions were 60 points in each dimension separated by 0.375 Å. The files were generated in PDBQT format. For the ligand, random starting positions and orientations were used. The Genetic Algorithm was used with 2,500,000 energy evaluations and a population of 300 individuals; 100 runs were carried out.

The molecular dynamics (MD) simulations were performed using Gromacs v4.5.5 (Pronk et al., 2013), with the Gromacs 96 (54a7) force field. The model structures of jmPE13 and the mutants were solvated with a three-point water model in a cubic box. Na^+^ and Cl^−^ ions were added to neutralize the charges in the system. Then, a steepest descent energy minimization was performed, followed by a 100 ps NVT and a 100 ps NPT equilibration at 300 K, and 10 ns MD simulations were performed at 300 K.

## 3 Results

### 3.1 Discovery of two potential plastic-degrading polyesterases

We previously isolated a bacterium strain *Pseudomonas* sp. JM16B3 from aquaculture water. To investigate the plastic-degrading ability of JM16B3, we treated semicrystalline PET and PBAT films with the fermentation supernatant of this strain. SEM observation showed that after 72 h of treatment at 30℃, both kinds of plastic films were damaged to a certain extent (Supplementary Figure S1), suggesting that there may be extracellular enzymes with polyester degrading activity. To discover potential polyester hydrolases, we blasted the genome of JM16B3 with the protein sequence of IsPETase. Two proteins were found to share 50% and 51% sequence identity with IsPETase and were named jmPE13 and jmPE14, respectively.

**FIGURE 1:**
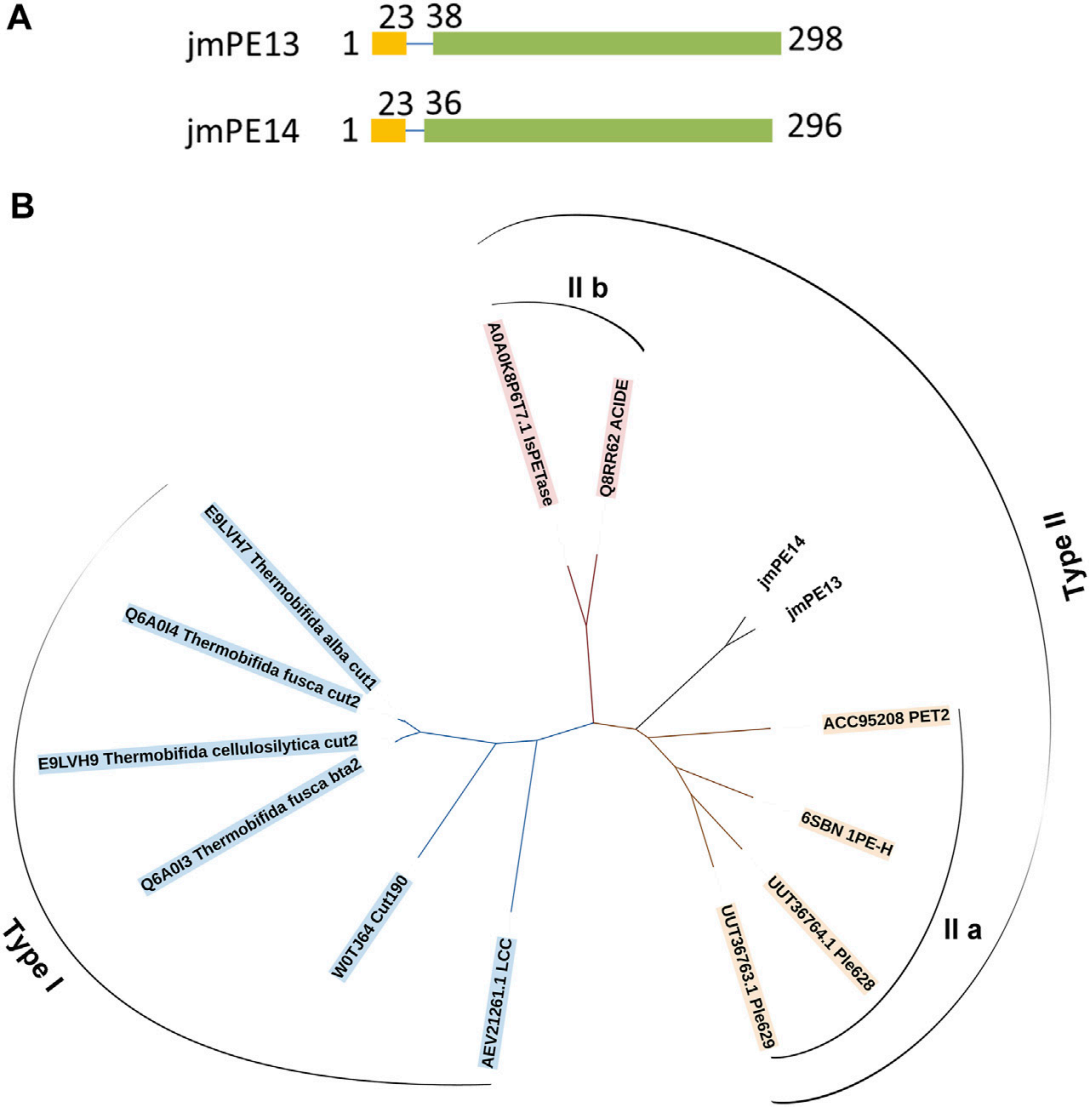
Domain composition and phylogenetic analysis of jmPE13 and jmPE14. **(A)** Schematic representation of the domain composition of jmPE13 and jmPE14. Signal peptide and α/β hydrolase fold domains are colored yellow and green, respectively. **(B)** Phylogenetic analysis of jmPE13, jmPE14, and the known PET-hydrolases. The accession numbers and the names of the selected PET-hydrolases are labeled.

**FIGURE 2:**
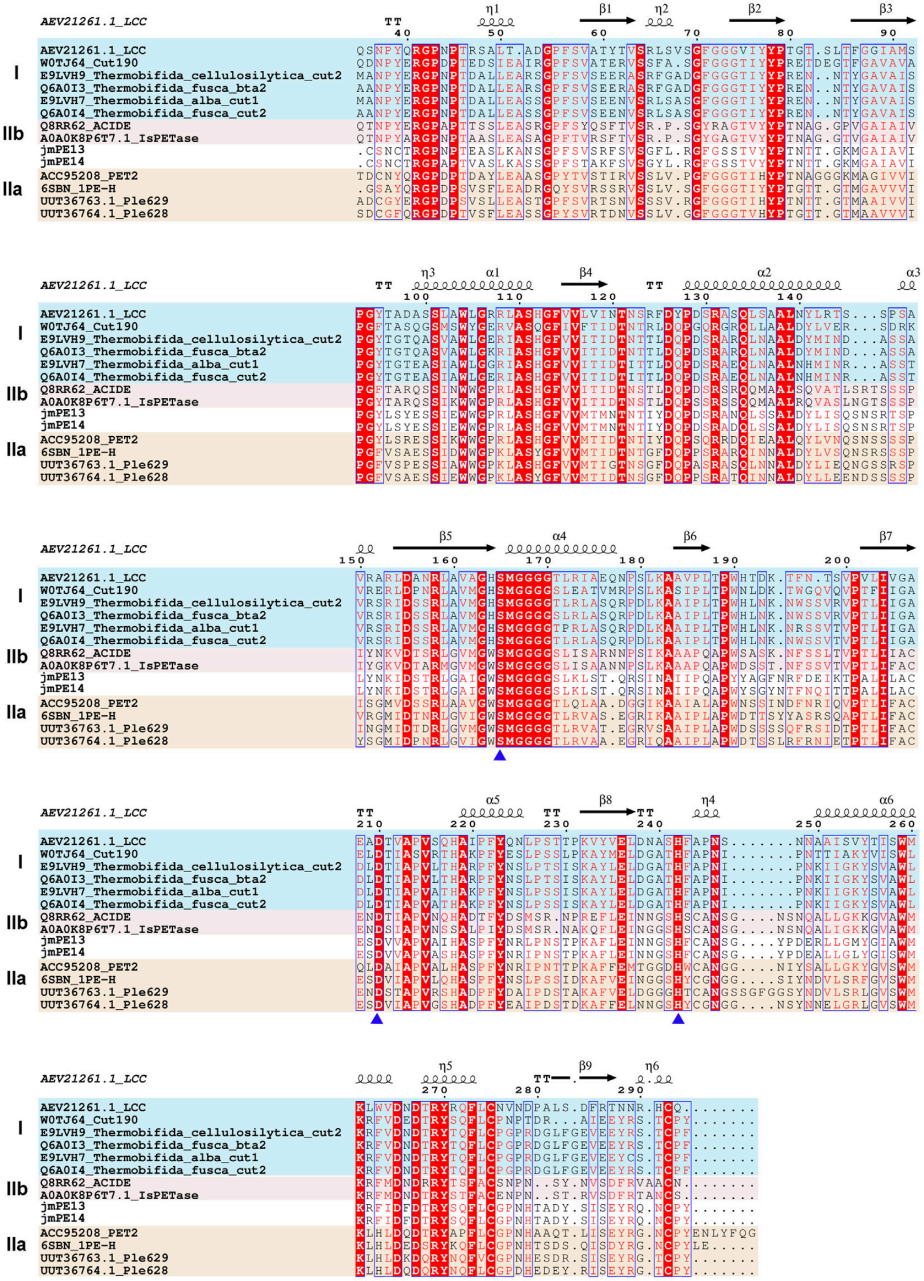
Multiple sequence alignment of jmPE13, jmPE14, and the characterized PET-hydrolases. The type I, type IIa, and type IIb enzymes are shown in blue, red, and orange background, respectively. The catalytic triads are indicated by blue triangles below.

There are 298 and 296 amino acids in jmPE13 and jmPE14, respectively, containing a signal peptide (amino acids 1-23) and a typical α/β hydrolase fold domain (Figure 1A). The protein sequences of jmPE13 and jmPE14 share 88% identity. Phylogenetic analysis showed that both jmPE13 and jmPE14 belong to type II PET-hydrolases (Figure 1B). Of the characterized enzymes, they showed the highest sequence identities to type IIa PET-hydrolases (53%–61%), while their protein sequence identities to type IIb and type I PET-hydrolases were 52%–53% and 46%–49%, respectively (Supplementary Table S2). Multiple sequence alignment showed that similar to the characterized type II PET-hydrolases, the loops after α2 and after β8 in jmPE13 and jmPE14 are longer than those of type I PET-hydrolases (Figure 2). JmPE13 and jmPE14 have the typical catalytic triads of the α/β hydrolase superfamily, which are S165-H243-D211 and S163-H241-D209, respectively.

### 3.2 Structural characteristics of jmPE13 and jmPE14

**FIGURE 3:**
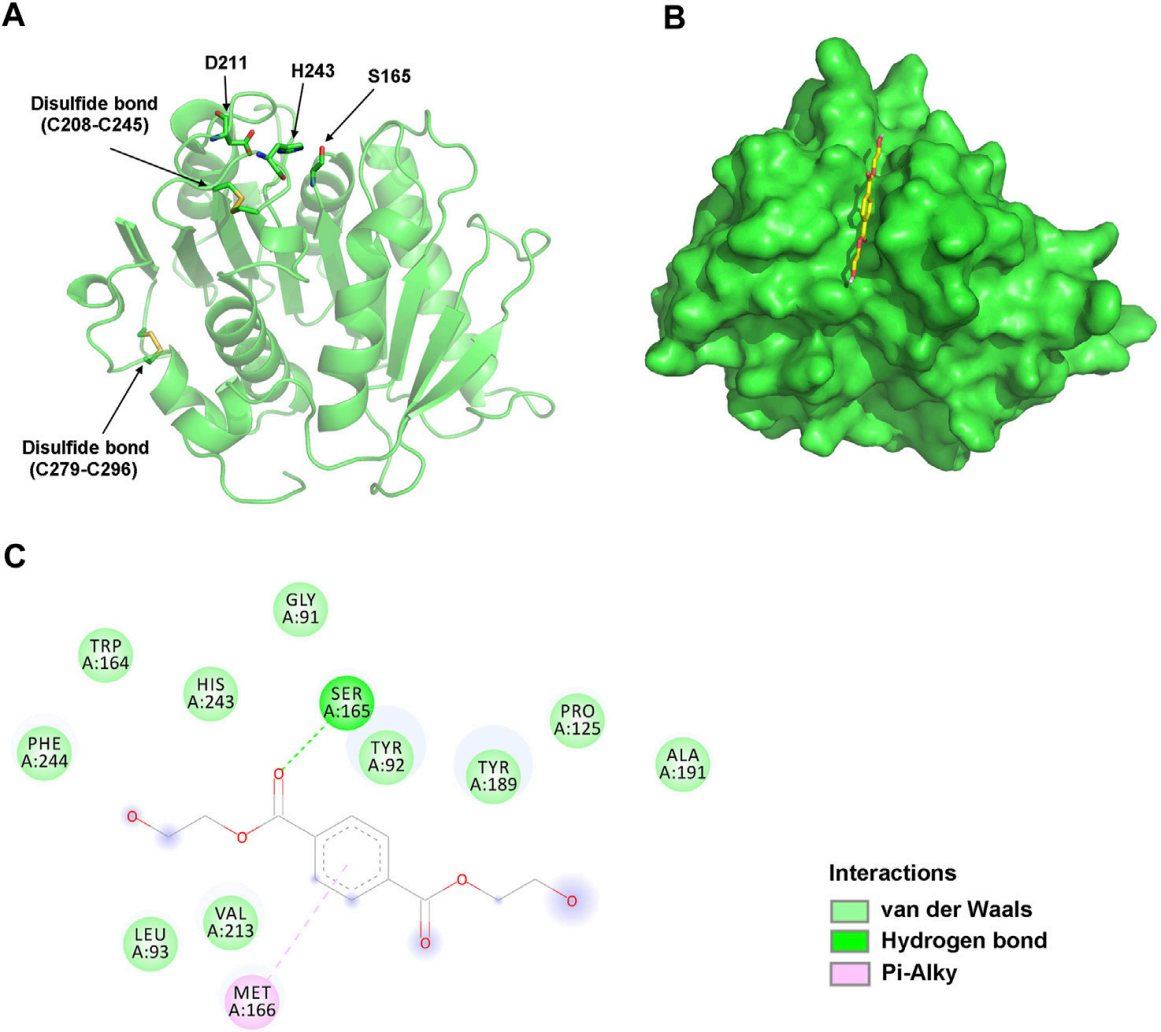
Structural analysis of jmPE13. **(A)** The overall model structure of jmPE13. The catalytic triads and the disulfide bonds are labeled and shown in sticks. **(B)** Binding mode of BHET to jmPE13 predicted by molecular docking. BHET is shown in yellow sticks. **(C)** 2D diagram of intermolecular interactions in the complex of jmPE13-BHET.

To analyze their structural characteristics, three-dimensional structural models of jmPE13 and jmPE14 were constructed using the crystal structure of PET2 (Nakamura et al., 2021) as a template. The models showed an α/β hydrolase fold with a nine-stranded β-sheet at the center surrounded by seven α-helices (Figure 3A; Supplementary Figure S2). Two disulfide bonds were formed in their structures, one near the active center, which is typical for type II PET-hydrolases, and the other at the C-terminus. A shallow cleft is formed on the molecular surface near the catalytic center of jmPE13 and jmPE14, which may be the substrate binding pocket.

To predict the possible binding modes of jmPE13 and jmPE14 to polyester plastic substrates, we performed molecular docking of the model structures with the PET model substrate BHET. As shown in Figure 3B, BHET could be well accommodated in the substrate-binding cleft. In the jmPE13-BHET complex, twelve residues (G91, Y92, L93, P125, W164, S165, M166, Y189, A191, V213, H243, and F244) formed intermolecular interactions with the substrate through hydrogen bonds, van der Waals, and Pi-Alkyl (Figure 3C). In jmPE14-BHET complex, the residues involved in substate-binding included Y90, Q122, P123, W162, S163, M164, W187, V211, H241, and F242 (Supplementary Figure S2).

### 3.3 Biochemical characterization of jmPE13 and jmPE14

**FIGURE 4:**
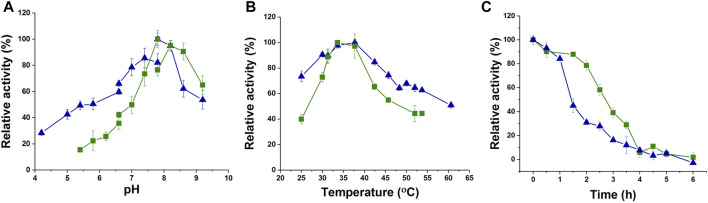
Biochemical characterization of jmPE13 and jmPE14. **(A)** pH-activity curves of jmPE13 and jmPE14. The citrate-sodium citrate buffer, phosphate buffer, and Tris-HCl buffer were used in the pH range 4.0–6.6, 6.6–7.8, and 7.8-9.2, respectively. **(B)** Temperature-activity curves of jmPE13 and jmPE14. **(C)** Thermal inactivation curves of jmPE13 and jmPE14 at 40°C. Green, jmPE13; blue, jmPE14.

To characterize the enzyme activity and catalytic properties of jmPE13 and jmPE14, we recombinantly expressed these two proteins. The genes of jmPE13 and jmPE14 (without the signal peptide) were cloned into the pET-32b vector and transformed into *E. coli* BL21 (DE3) for expression of the proteins. After nickel affinity chromatography, purified proteins were obtained (Supplementary Figure S3). To determine their optimal catalytic conditions, we examined the effects of pH and temperature on the enzyme activities of jmPE13 and jmPE14. As shown in Figure 4A, B, their optimal pH was about 7.8–8.0, and the optimal temperature was about 34°C–37°C. In the range of pH 7.5-8.5 and temperature 30°C–40°C, both jmPE13 and jmPE14 can maintain more than 70% of the highest enzyme activity. To investigate the thermal stability of these two enzymes, the enzyme proteins were incubated at 40°C and the residual activities were examined. The thermal inactivation curves showed that jmPE13 could maintain more than 70% of the enzyme activity within 2 h of incubation, then decreased to 50% at about 2.5 h, and almost completely lost the enzyme activity after incubation for more than 6 h (Figure 4C). The thermal stability of jmPE14 was slightly lower than that of jmPE13. Although it maintained more than 80% activity within 1 h, it rapidly dropped to less than 50% after 1.5 h incubation.

### 3.4 Polyester hydrolyzing activity of jmPE13 and jmPE14

**FIGURE 5:**
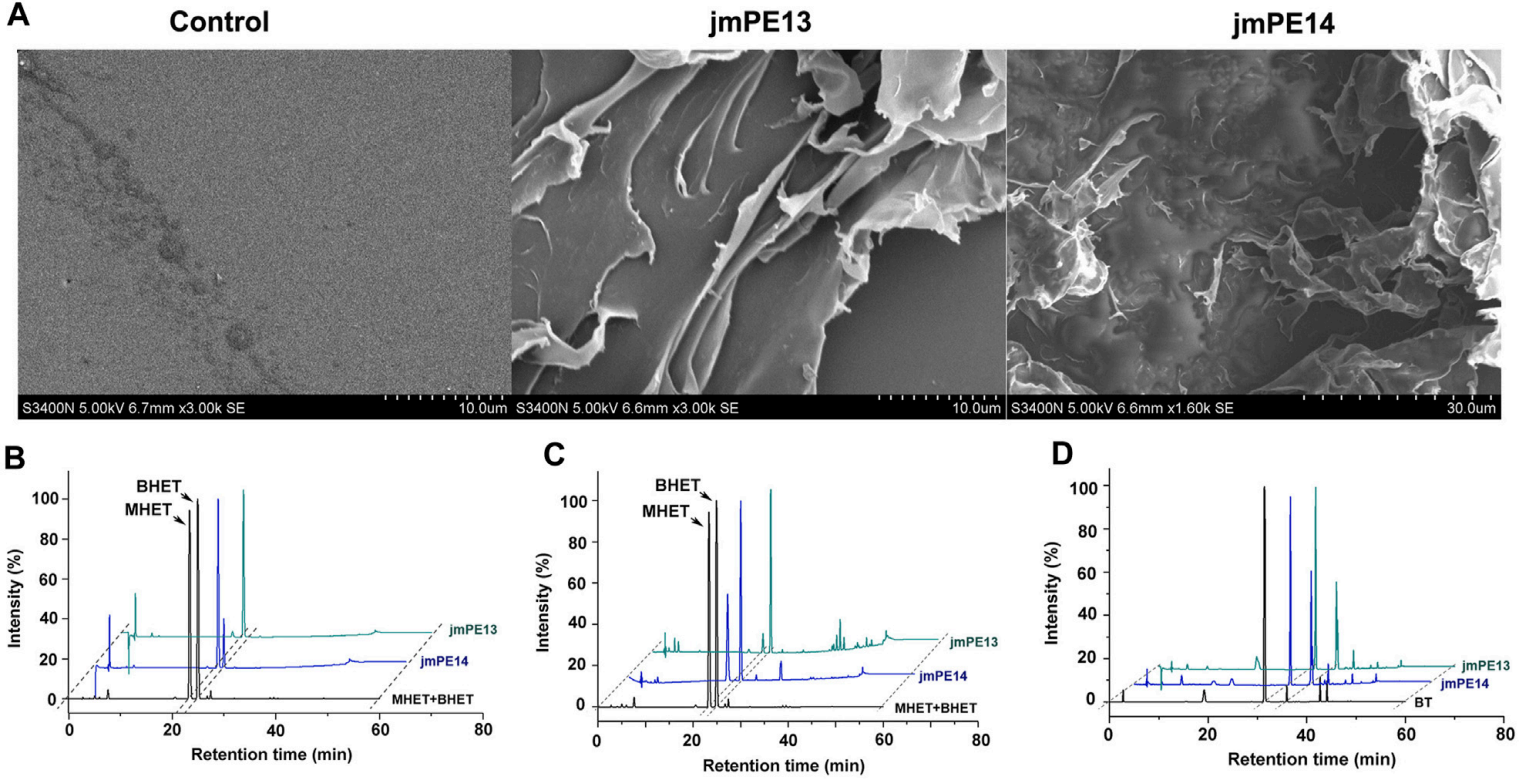
Polyester hydrolytic activity of jmPE13 and jmPE14. **(A)** SEM observation of the post-consumer PET bottles treated by jmPE13 and jmPE14 at 30°C for 7 days. **(B–D)** HPLC analysis of BHET-, PET-, and PBAT-hydrolytic activity of jmPE13 and jmPE14.

To investigate the polyester hydrolyzing activity, purified proteins of jmPE13 and jmPE14 were incubated with the slices of post-consumer PET bottles at 30°C for 7 days. The surface morphology of the PET slices was observed by SEM. Compared with the buffer control group, the surface of the PET slices was significantly damaged after being treated with jmPE13 and jmPE14 (Figure 5A). We further tested the hydrolytic activity of jmPE13 and jmPE14 on PET and its derived oligomer BHET by HPLC. The results showed that after 5 h of incubation at 30°C, jmPE13 could completely convert BHET to MHET, while jmPE14 was slightly less active and a small amount of BHET remained (Figure 5B). For semicrystalline PET microplastics, the reaction was performed at 30°C for 90 h. JmPE13 and jmPE14 hydrolyzed PET to produce BHET and MHET (Figure 5C). Since some PET hydrolases have been reported to hydrolyze PBAT, we also examined the hydrolyzing activity of jmPE13 and jmPE14 on PBAT. The results showed that, after incubation at 30°C for 48 h, both enzymes could hydrolyze PBAT to produce BT (Figure 5D). These results indicate that jmPE13 and jmPE14 have hydrolytic activity on PET and PBAT polyesters.

### 3.5 Enzyme engineering of jmPE13

**FIGURE 6:**
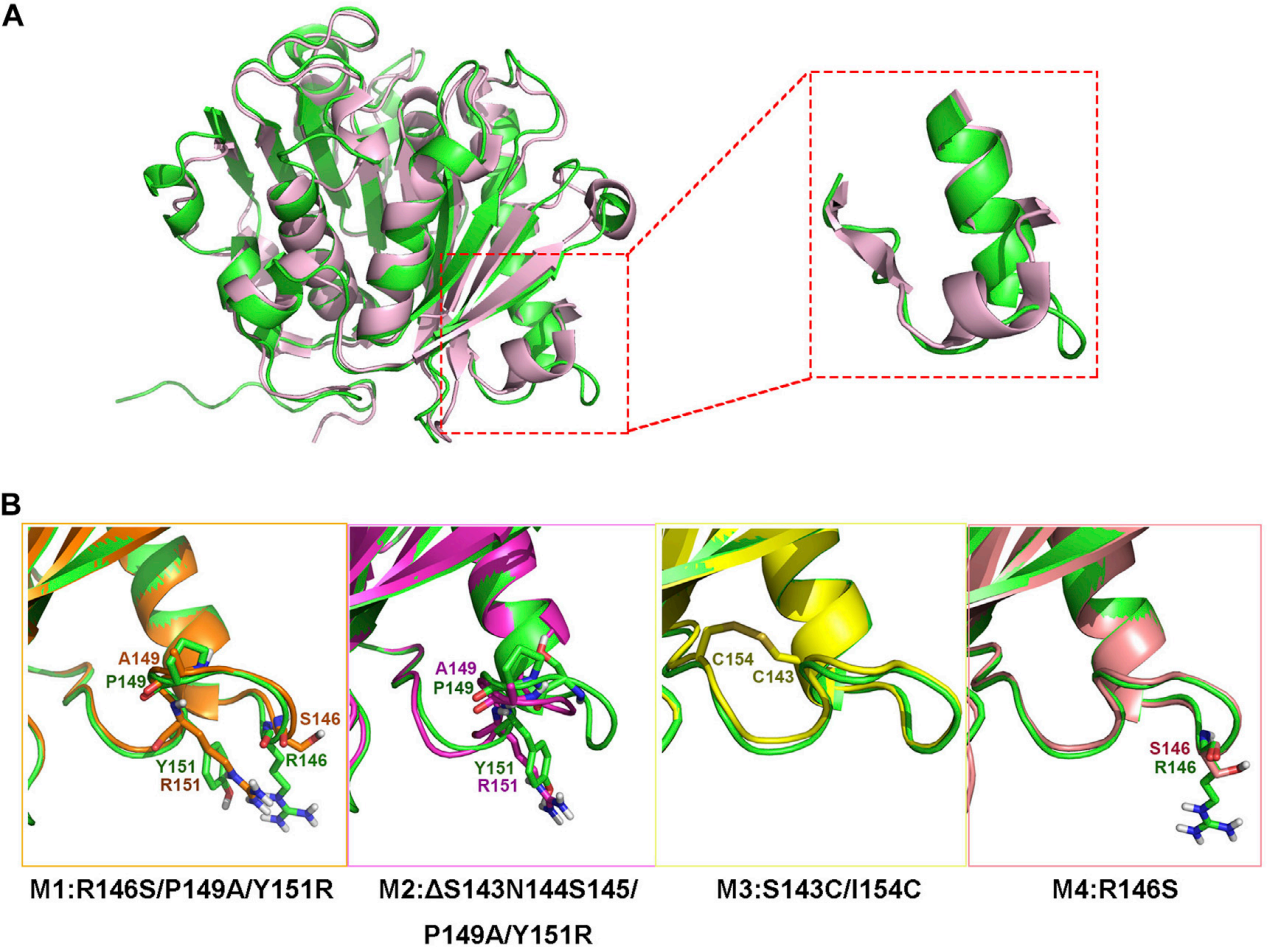
Design of the mutants. **(A)** Superposition of the model structure of jmPE13 (green) and the structure of LCC^ICCG^ (pink). The loops after α2 are circled and shown in the enlarged diagram. **(B)** Mutations introduced in the individual mutants. The mutated sites are labeled and shown in sticks.

**FIGURE 7:**
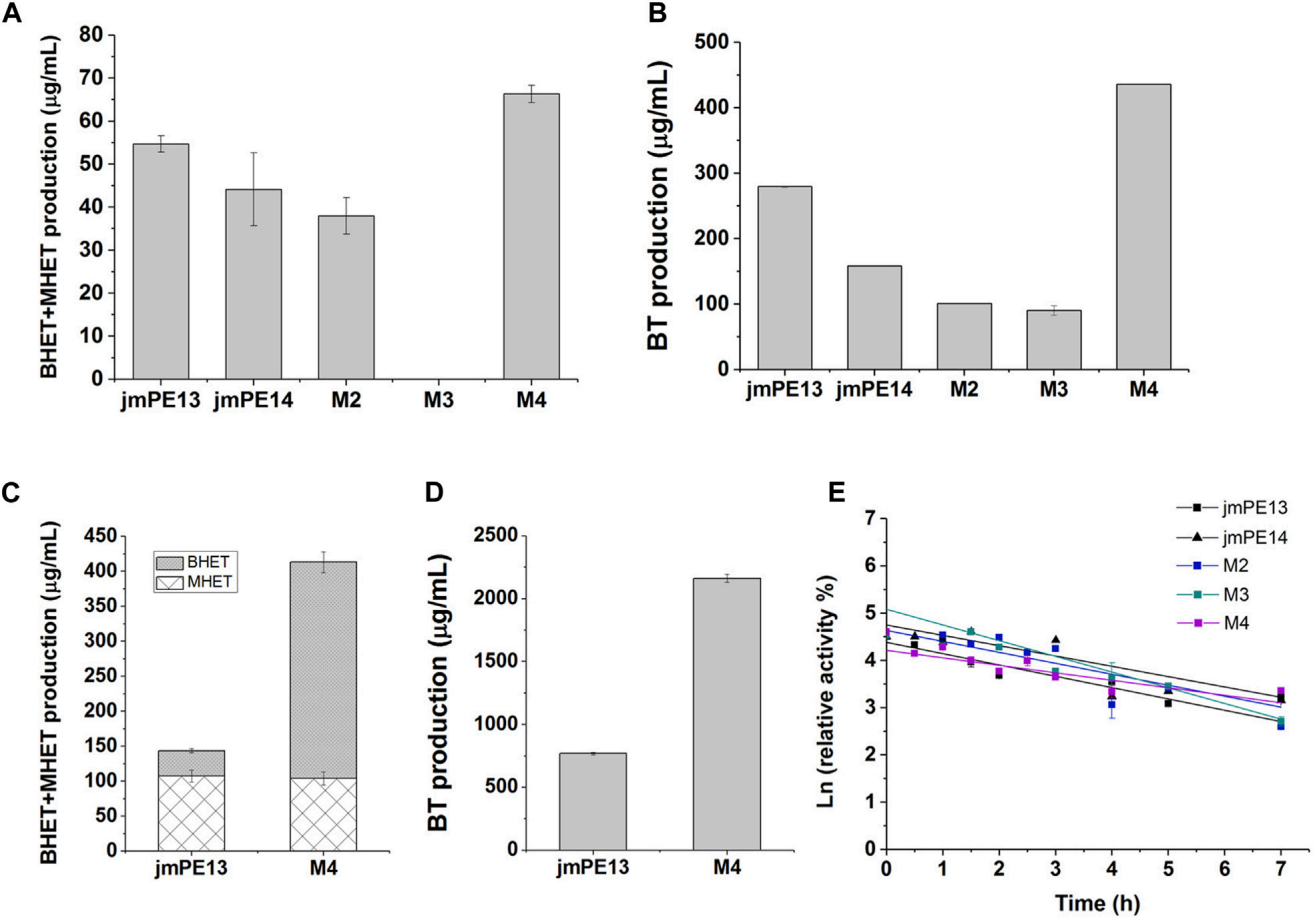
Enzyme activity and thermal stability of the mutants. **(A)** PET-hydrolytic activity of the enzymes. The reaction mixtures were incubated at 30°C for 24 h, and the produced MHET and BHET were measured by HPLC. **(B)** The PBAT-hydrolytic activity of the enzymes characterized by measuring the produced BT. The reaction mixtures were incubated at 30°C for 24 h. **(C,D)** are the activity of jmPE13 and M4 for PET and PBAT, respectively, reacting at 30°C for 90 h. **(E)** Thermal inactivation of the enzymes at 40°C. Proteins were incubated at 40°C and sampled at regular intervals to determine residual activity against PBAT at 30°C.

Although jmPE13 and jmPE14 can hydrolyze polyester plastics, their hydrolysis activity and thermal stability are still very low (Figures 4, 7). To find sites for modification to improve its enzyme activity, we performed a structural alignment between jmPE13 and the known PET hydrolase mutant LCC^ICCG^ (PDB entry: 6THT) (Tournier et al., 2020). As shown in Figure 6A, a significant difference is that the α2 of jmPE13 is followed by a longer loop, while the corresponding position of LCC^ICCG^ is an α-helix, which is consistent with the results of multiple sequence alignment (Figure 2). We targeted this loop and designed four mutants referring to the structure of LCC^ICCG^ (Figure 6B). In mutants M1 (R146S/P149A/Y151R) and M4 (R146S), the selected sites were mutated to the corresponding residues of LCC^ICCG^, and a truncated mutant M2 (ΔS143N144S145/P149A/Y151R) was constructed based on M1. In addition, we tried to introduce a disulfide bond to reinforce this loop (M3: S143C/I154C). All mutants except M1 were solubly expressed in *E. coli.* (Supplementary Figure S3).

We examined the enzyme activity of the mutants, but unfortunately, both M2 and M3 showed varying degrees of decreased hydrolytic activity for PET and PBAT compared to jmPE13 (Figures 7A, B). M3 was almost completely inactive on PET. However, M4 showed a significant increase in hydrolytic activity for both polyesters. Moreover, this increase in activity became more pronounced when the reaction time was prolonged. After the reaction at 30°C for 90 h, the activity of M4 against both PET and PBAT reached about 3 times that of jmPE13 (Figures 7C, D). The improvement of PET hydrolytic activity of M4 was mainly due to the increase of BHET products, while the production of MHET was comparable to that of jmPE13. The enzymatic kinetic parameters of jmPE13 and M4 on BHET were determined. The maximum reaction rate (V_max_) of M4 (5.22 ± 0.17 μM/min) was higher than that of jmPE13 (4.09 ± 0.5 μM/min); however, the *K*_m_ value of M4 (0.58 ± 0.05 mM) for BHET was also higher than that of jmPE13 (0.39 ± 0.07 mM), indicating that the increased activity of the mutant was accompanied by a decrease in its affinity for this small substrate. To investigate the thermostability of the mutants, each enzyme protein was incubated at 40°C for a certain time and their residual activity against PBAT was examined (Figure 7E; Table 2). The results showed no significant change in the thermal stability of M2 and M3 relative to the wild-type, with a slight increase and decrease, respectively. However, the thermal stability of M4 has been significantly improved, and its thermal inactivation half-life at 40°C is about 1.5 h longer than that of jmPE13, which is about 1.5 times that of jmPE13.

**TABLE 2:** Thermodynamic parameters of the enzymes.

Enzymes	jmPE13	jmPE14	M2	M3	M4
*k*_*inact*_	0.2396	0.2356	0.2318	0.3323	0.1584
*t*_*1/2*_ at 40°C (h)	2.89	2.94	2.99	2.09	4.37

**FIGURE 8:**
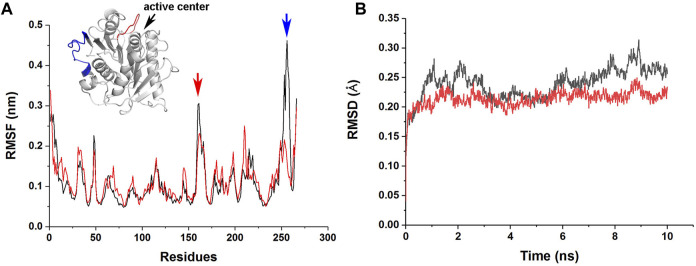
MD simulations of jmPE13 and M4. **(A)** RMSF of jmPE13 (black) and M4 (red) in the 10 ns MD simulations. Two loops with large RMSF variations are shown in the embedded structural diagram. The blue and red arrows indicate the corresponding loops. **(B)** RMSD of jmPE13 (black) and M4 (red).

To understand the molecular basis of the enhanced stability and enzymatic activity of M4 by the R146S single point mutation, we performed MD simulations of jmPE13 and M4. The results showed that this mutation resulted in a decrease in root mean square fluctuation (RMSF) of a loop near the catalytic center and a C-terminal loop, indicating a decrease in the flexibility of these two regions (Figure 8A). The overall rigidity of the molecule was also increased as indicated by the change in root mean square deviation (RMSD) (Figure 8B). We speculate that the increased activity of M4 may also be due to its improved stability, which makes its activity decline more slowly during the reaction period. This is also consistent with the phenomenon that the increase in activity is more pronounced with prolonged reaction time.

## 4 Discussion

In this study, we discovered and biochemically characterized two polyester hydrolases derived from our previously isolated *Pseudomonas* strain. Both jmPE13 and jmPE14 exhibited the ability to catalyze the hydrolysis of post-consumer PET plastic bottles, amorphous PET, the PET derivative BHET, and PBAT at moderate temperatures. For each of these polyester substrates, jmPE13 showed higher activity than jmPE14. This phenomenon of coexistent tandem enzymes with different activities in the same strain may be ubiquitous in nature. For example, the two cutinases Thc_Cut1 and Thc_Cut2 from *Thermobifida cellulosilytica* differ in only 18 amino acids. However, the difference of amino acids near the active center leads to the difference of substrate binding cleft structure, and then leads to the difference of their hydrolysis activities to long-chain aliphatic substrates and PET (Arnling Baath et al., 2022). There are 20 differential amino acids between jmPE13 and jmPE14, most of which are distributed on the enzyme surface (Supplementary Figure S4). In particular, residues located near the active center (Y189 and F193 of jmPE13; W189 and Y191 of jmPE14), which are the parts constituting the substrate binding clefts, may affect substrate binding and become the main factor causing the different activities of the two enzymes. JmPE13 and jmPE14 hydrolyze PET yielding BHET and MHET, and hydrolyze PBAT resulting in BT formation. Some enzymes have been reported to further hydrolyze MHET and BT to TPA with extended reaction time, such as Ple629 (Meyer Cifuentes et al., 2022). This activity is absent in jmPE13 and jmPE14, but their hydrolytic activity for PET and PBAT is relatively higher, suggesting that they may be more suitable for catalyzing hydrolysis of highly polymeric substrates.

Activity and stability are the most important properties of industrial enzymes and the major goals of enzyme engineering. We modified jmPE13 by referring to the structure of a representative enzyme LCC^ICCG^. MD simulations showed that all three designed mutants improved the overall molecular rigidity (Supplementary Figure S5). The local structure of the mutated regions of M3 and M4 did not change significantly, except for the truncated mutation that caused the corresponding loop of M2 to become shorter (Supplementary Figure S5C). However, they all lead to conformational changes of the distant loops, especially those located near the active center. The changes of M2 and M3 are more obvious, the loop near their C-terminus became a helix. And these larger changes may also be responsible for their decreased activity. A deeper understanding of the structure-function relationships of these enzymes in future is needed for better rational design.

## 5 Conclusion

In summary, two polyester plastic hydrolases, jmPE13 and jmPE14, were identified in a *Pseudomonas* strain. The enzyme proteins were expressed and purified in *E. coli*, and their enzyme properties were characterized. Both jmPE13 and jmPE14 showed hydrolytic activity towards PET and PBAT polyester plastics. JmPE13 was further modified by enzyme engineering, and a mutant M4 with improved enzyme activity and thermal stability was obtained. The results of this study provide a basis for further research and molecular design of polyester plastic hydrolases.

## Data Availability

The original contributions presented in the study are included in the article/Supplementary Material, further inquiries can be directed to the corresponding author.

## References

[B1] Ali S. S. Elsamahy T. Al-Tohamy R. Zhu D. Mahmoud Y. A. Koutra E. (2021). Plastic wastes biodegradation: Mechanisms, challenges and future prospects. Sci. Total Environ. 780, 146590. 10.1016/j.scitotenv.2021.146590 34030345

[B2] Arnling Baath J. Novy V. Carneiro L. V. Guebitz G. M. Olsson L. Westh P. (2022). Structure-function analysis of two closely related cutinases from Thermobifida cellulosilytica. Biotechnol. Bioeng. 119 (2), 470–481. 10.1002/bit.27984 34755331 PMC9299132

[B3] Blazquez-Sanchez P. Vargas J. A. Furtado A. A. Grinen A. Leonardo D. A. Sculaccio S. A. (2023). Engineering the catalytic activity of an Antarctic PET-degrading enzyme by loop exchange. Protein Sci. 32 (9), e4757. 10.1002/pro.4757 37574805 PMC10464292

[B4] Chen S. Tong X. Woodard R. W. Du G. Wu J. Chen J. (2008). Identification and characterization of bacterial cutinase. J. Biol. Chem. 283 (38), 25854–25862. 10.1074/jbc.M800848200 18658138 PMC3258855

[B5] Cui Y. Chen Y. Liu X. Dong S. Tian Y. e. Qiao Y. (2021). Computational redesign of a PETase for plastic biodegradation under ambient condition by the GRAPE strategy. ACS Catal. 11 (3), 1340–1350. 10.1021/acscatal.0c05126

[B6] Gouet P. Courcelle E. Stuart D. I. Metoz F. (1999). ESPript: analysis of multiple sequence alignments in PostScript. Bioinformatics 15 (4), 305–308. 10.1093/bioinformatics/15.4.305 10320398

[B7] Jia Y. Samak N. A. Hao X. Chen Z. Wen Q. Xing J. (2022). Hydrophobic cell surface display system of PETase as a sustainable biocatalyst for PET degradation. Front. Microbiol. 13, 1005480. 10.3389/fmicb.2022.1005480 36246227 PMC9559558

[B8] Jia Y. P. Samak N. A. Hao X. M. Chen Z. Yang G. M. Zhao X. H. (2021). Nano-immobilization of PETase enzyme for enhanced polyethylene terephthalate biodegradation. Biochem. Eng. J. 176, 108205. 10.1016/j.bej.2021.108205

[B9] Jimenez D. J. Ozturk B. Wei R. Bugg T. D. Amaya Gomez C. V. Salcedo Galan F. (2022). Merging plastics, microbes, and enzymes: highlights from an international workshop. Appl. Environ. Microbiol. 88 (14), 10.1128/aem.00721-22 PMC9317848 35762791

[B10] Kumar S. Stecher G. Li M. Knyaz C. Tamura K. (2018). MEGA X: molecular evolutionary genetics analysis across computing platforms. Mol. Biol. Evol. 35 (6), 1547–1549. 10.1093/molbev/msy096 29722887 PMC5967553

[B11] Letunic I. Bork P. (2007). Interactive Tree of Life (iTOL): an online tool for phylogenetic tree display and annotation. Bioinformatics 23 (1), 127–128. 10.1093/bioinformatics/btl529 17050570

[B12] Li A. Sheng Y. Cui H. Wang M. Wu L. Song Y. (2023). Discovery and mechanism-guided engineering of BHET hydrolases for improved PET recycling and upcycling. Nat. Commun. 14 (1), 4169. 10.1038/s41467-023-39929-w 37443360 PMC10344914

[B13] Li Z. Zhao Y. Wu P. Wang H. Li Q. Gao J. (2022). Structural insight and engineering of a plastic degrading hydrolase Ple629. Biochem. Biophys. Res. Commun. 626, 100–106. 10.1016/j.bbrc.2022.07.103 35981419

[B14] Liu F. Wang T. Yang W. Zhang Y. Gong Y. Fan X. (2023). Current advances in the structural biology and molecular engineering of PETase. Front. Bioeng. Biotechnol. 11, 1263996. 10.3389/fbioe.2023.1263996 37795175 PMC10546322

[B15] Lu H. Diaz D. J. Czarnecki N. J. Zhu C. Kim W. Shroff R. (2022). Machine learning-aided engineering of hydrolases for PET depolymerization. Nature 604 (7907), 662–667. 10.1038/s41586-022-04599-z 35478237

[B16] MacLeod M. Arp H. P. H. Tekman M. B. Jahnke A. (2021). The global threat from plastic pollution. Science 373 (6550), 61–65. 10.1126/science.abg5433 34210878

[B17] Meyer Cifuentes I. E. Wu P. Zhao Y. Liu W. Neumann-Schaal M. Pfaff L. (2022). Molecular and biochemical differences of the tandem and cold-adapted pet hydrolases Ple628 and Ple629, isolated from a marine microbial consortium. Front. Bioeng. Biotechnol. 10, 930140. 10.3389/fbioe.2022.930140 35935485 PMC9350882

[B18] Morris G. M. Huey R. Lindstrom W. Sanner M. F. Belew R. K. Goodsell D. S. (2009). AutoDock4 and AutoDockTools4: automated docking with selective receptor flexibility. J. Comput. Chem. 30 (16), 2785–2791. 10.1002/jcc.21256 19399780 PMC2760638

[B19] Nakamura A. Kobayashi N. Koga N. Iino R. (2021). Positive charge introduction on the surface of thermostabilized PET hydrolase facilitates PET binding and degradation. ACS Catal. 11 (14), 8550–8564. 10.1021/acscatal.1c01204

[B20] Palm G. J. Reisky L. Bottcher D. Muller H. Michels E. A. P. Walczak M. C. (2019). Structure of the plastic-degrading Ideonella sakaiensis MHETase bound to a substrate. Nat. Commun. 10 (1), 1717. 10.1038/s41467-019-09326-3 30979881 PMC6461665

[B21] Pronk S. Pall S. Schulz R. Larsson P. Bjelkmar P. Apostolov R. (2013). GROMACS 4.5: a high-throughput and highly parallel open source molecular simulation toolkit. Bioinformatics 29 (7), 845–854. 10.1093/bioinformatics/btt055 23407358 PMC3605599

[B22] Qiu J. Chen Y. Zhang L. Wu J. Zeng X. Shi X. (2023). A comprehensive review on enzymatic biodegradation of polyethylene terephthalate. Environ. Res. 240, 117427. 10.1016/j.envres.2023.117427 37865324

[B23] Roth C. Wei R. Oeser T. Then J. Follner C. Zimmermann W. (2014). Structural and functional studies on a thermostable polyethylene terephthalate degrading hydrolase from Thermobifida fusca. Appl. Microbiol. Biotechnol. 98 (18), 7815–7823. 10.1007/s00253-014-5672-0 24728714

[B24] Samak N. A. Jia Y. Sharshar M. M. Mu T. Yang M. Peh S. (2020). Recent advances in biocatalysts engineering for polyethylene terephthalate plastic waste green recycling. Environ. Int. 145, 106144. 10.1016/j.envint.2020.106144 32987219

[B25] Shi L. Liu P. Tan Z. Zhao W. Gao J. Gu Q. (2023). Complete depolymerization of PET wastes by an evolved PET hydrolase from directed evolution. Angew. Chem. Int. Ed. Engl. 62 (14), 10.1002/anie.202218390 36751696

[B26] Sievers F. Higgins D. G. (2018). Clustal Omega for making accurate alignments of many protein sequences. Protein Sci. 27 (1), 135–145. 10.1002/pro.3290 28884485 PMC5734385

[B27] Sui B. Wang T. Fang J. Hou Z. Shu T. Lu Z. (2023). Recent advances in the biodegradation of polyethylene terephthalate with cutinase-like enzymes. Front. Microbiol. 14, 1265139. 10.3389/fmicb.2023.1265139 37849919 PMC10577388

[B28] Tamoor M. Samak N. A. Jia Y. Mushtaq M. U. Sher H. Bibi M. (2021). Potential use of microbial enzymes for the conversion of plastic waste into value-added products: a viable solution. Front. Microbiol. 12, 777727. 10.3389/fmicb.2021.777727 34917057 PMC8670383

[B29] Tournier V. Duquesne S. Guillamot F. Cramail H. Taton D. Marty A. (2023). Enzymes' power for plastics degradation. Chem. Rev. 123 (9), 5612–5701. 10.1021/acs.chemrev.2c00644 36916764

[B30] Tournier V. Topham C. M. Gilles A. David B. Folgoas C. Moya-Leclair E. (2020). An engineered PET depolymerase to break down and recycle plastic bottles. Nature 580 (7802), 216–219. 10.1038/s41586-020-2149-4 32269349

[B31] Wallace P. W. Haernvall K. Ribitsch D. Zitzenbacher S. Schittmayer M. Steinkellner G. (2017). PpEst is a novel PBAT degrading polyesterase identified by proteomic screening of *Pseudomonas pseudoalcaligenes* . Appl. Microbiol. Biotechnol. 101 (6), 2291–2303. 10.1007/s00253-016-7992-8 27872998 PMC5320007

[B32] Waterhouse A. Bertoni M. Bienert S. Studer G. Tauriello G. Gumienny R. (2018). SWISS-MODEL: homology modelling of protein structures and complexes. Nucleic Acids Res. 46 (W1), W296–W303. 10.1093/nar/gky427 29788355 PMC6030848

[B33] Wei R. von Haugwitz G. Pfaff L. Mican J. Badenhorst C. P. S. Liu W. (2022). Mechanism-based design of efficient PET hydrolases. ACS Catal. 12 (6), 3382–3396. 10.1021/acscatal.1c05856 35368328 PMC8939324

[B34] Xu A. Zhou J. Blank L. M. Jiang M. (2023). Future focuses of enzymatic plastic degradation. Trends Microbiol. 31 (7), 668–671. 10.1016/j.tim.2023.04.002 37121829

[B35] Yang Y. Min J. Xue T. Jiang P. Liu X. Peng R. (2023). Complete bio-degradation of poly(butylene adipate-co-terephthalate) via engineered cutinases. Nat. Commun. 14 (1), 1645. 10.1038/s41467-023-37374-3 36964144 PMC10039075

[B36] Yoshida S. Hiraga K. Takehana T. Taniguchi I. Yamaji H. Maeda Y. (2016). A bacterium that degrades and assimilates poly(ethylene terephthalate). Science 351 (6278), 1196–1199. 10.1126/science.aad6359 26965627

[B37] Zhu B. Wang D. Wei N. (2022). Enzyme discovery and engineering for sustainable plastic recycling. Trends Biotechnol. 40 (1), 22–37. 10.1016/j.tibtech.2021.02.008 33676748

