# Supplementary material for: Characterization and engineering of plastic-degrading polyesterases jmPE13 and jmPE14 from Pseudomonas bacterium
Source: Front Bioeng Biotechnol. 2024 Feb 14;12:1349010. doi: 10.3389/fbioe.2024.1349010 (PMC10904013; doi:10.3389/fbioe.2024.1349010)
Supplement: Supplementary file 1 [file DataSheet1.docx]

Supplementary Material

# Supplementary Figures and Tables

## Supplementary Figures


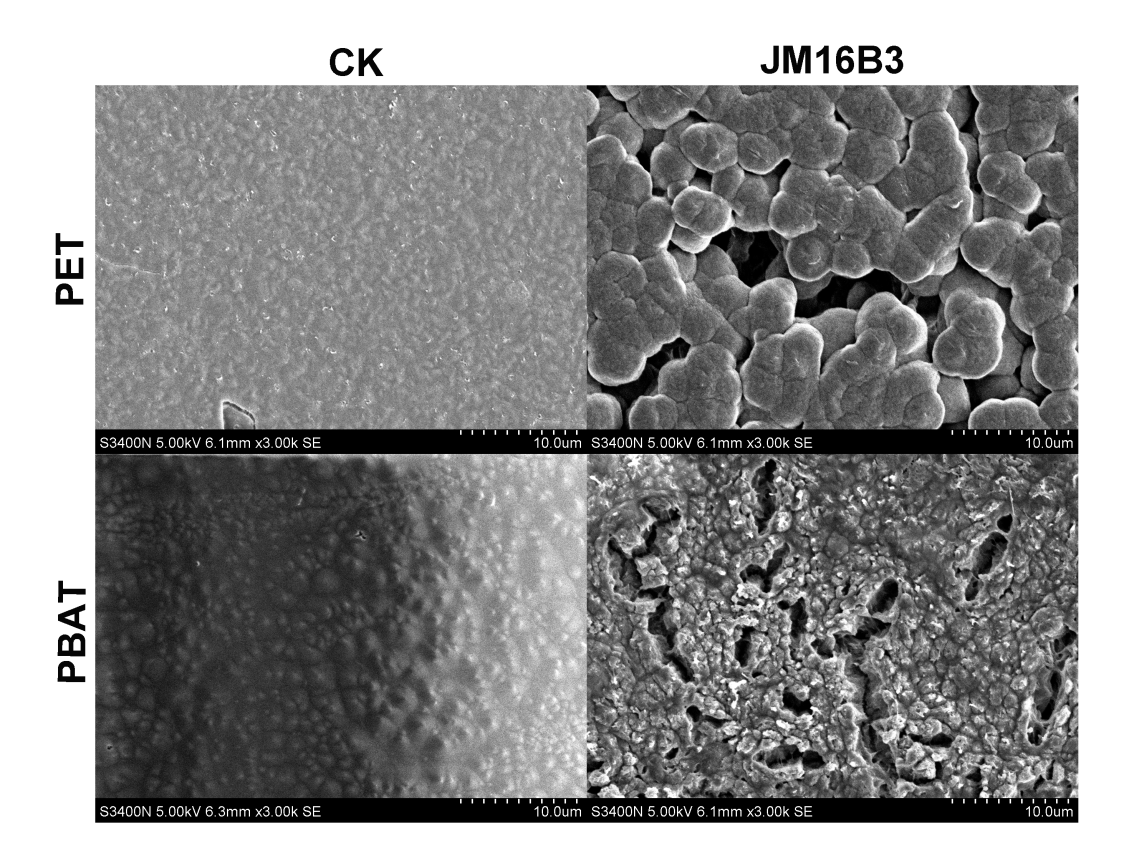


**Supplementary Figure 1.** SEM observation of the semicrystalline PET and PBAT films treated by the fermentation supernatant of JM16B3 at 30 °C for 72 hours.


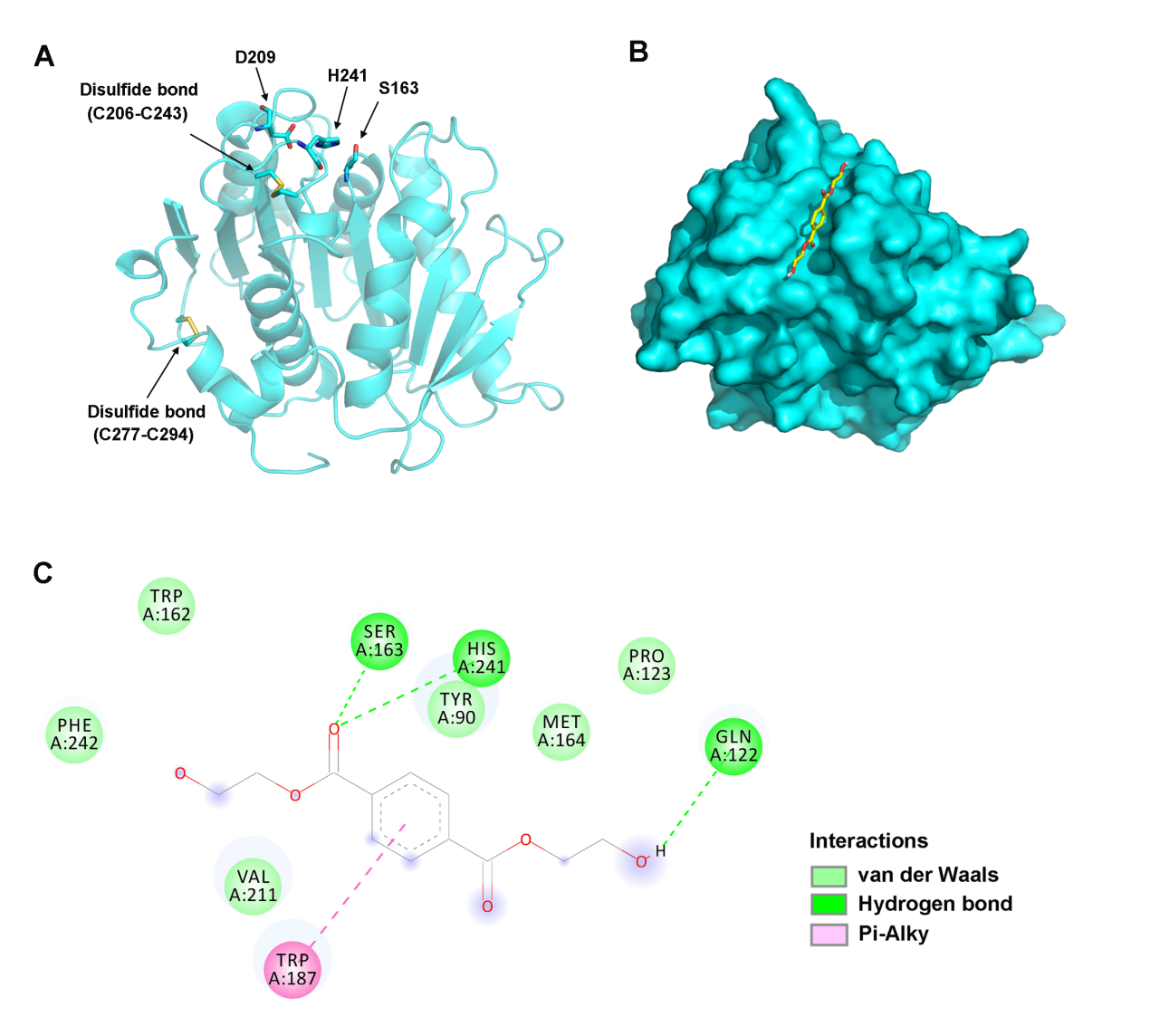


**Supplementary Figure 2.** Structural analysis of jmPE14. (A) the overall model structure of jmPE14. The catalytic triads and the disulfide bonds are labeled and shown in sticks. (B) Binding mode of BHET to jmPE14 predicted by molecular docking. BHET is shown in yellow sticks. (C) 2D diagram of intermolecular interactions in the complex of jmPE14-BHET.


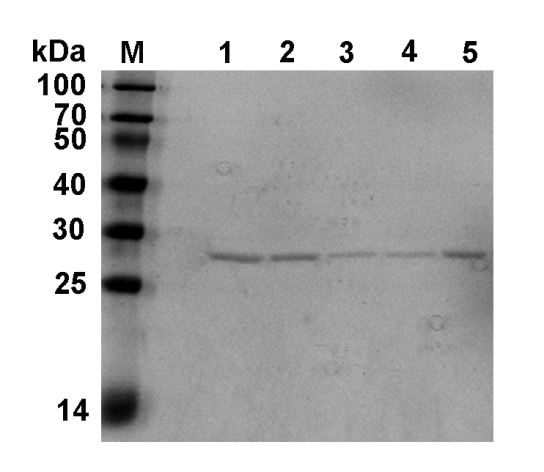


**Supplementary Figure 3.** SDS-PAGE analysis of the purified proteins of the enzymes. M, protein markers; lane 1, jmPE13; lane 2, jmPE14; lane 3, M2; lane 4, M3; lane 5, M4.


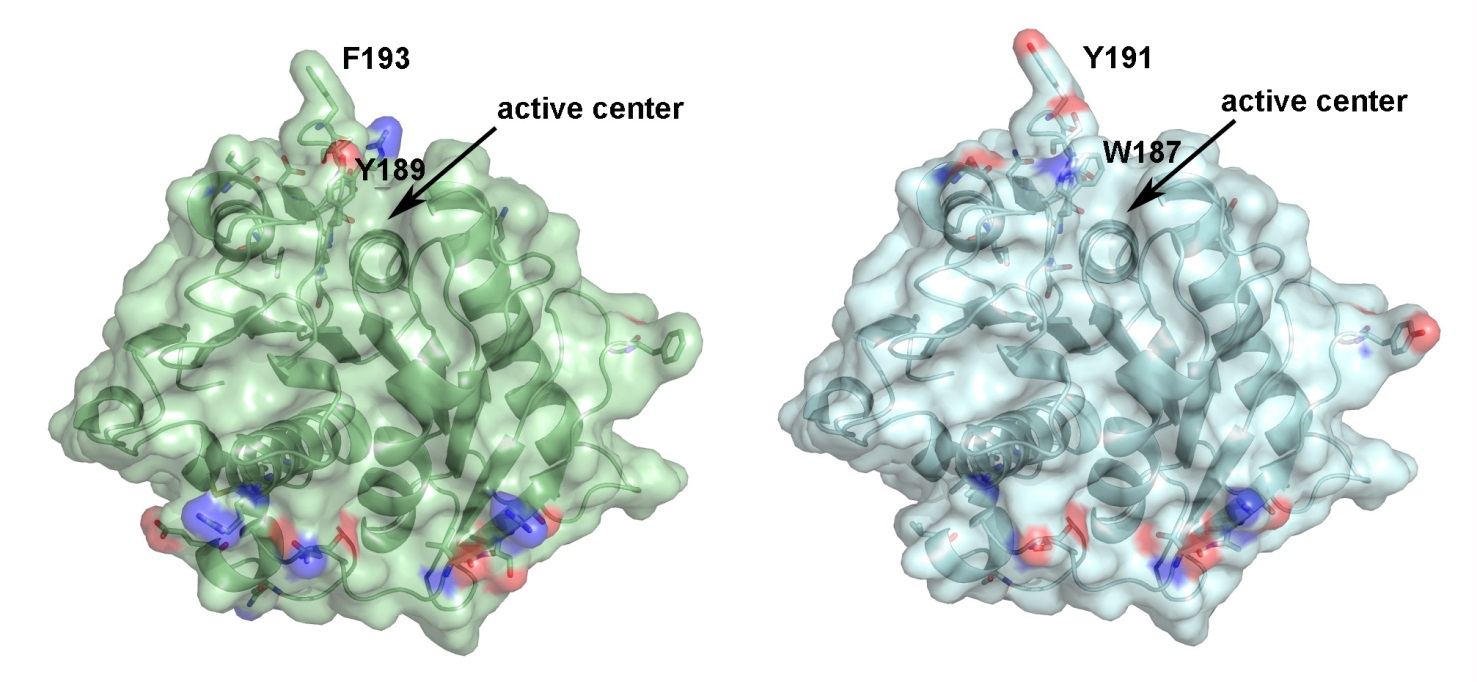


**Supplementary Figure 4.** Schematic representation of different amino acids of jmPE13 (green) and jmPE14 (cyan). The differential amino acids are shown in sticks, the active centers are indicated with arrows, and the differential amino acids near the active centers are labeled.


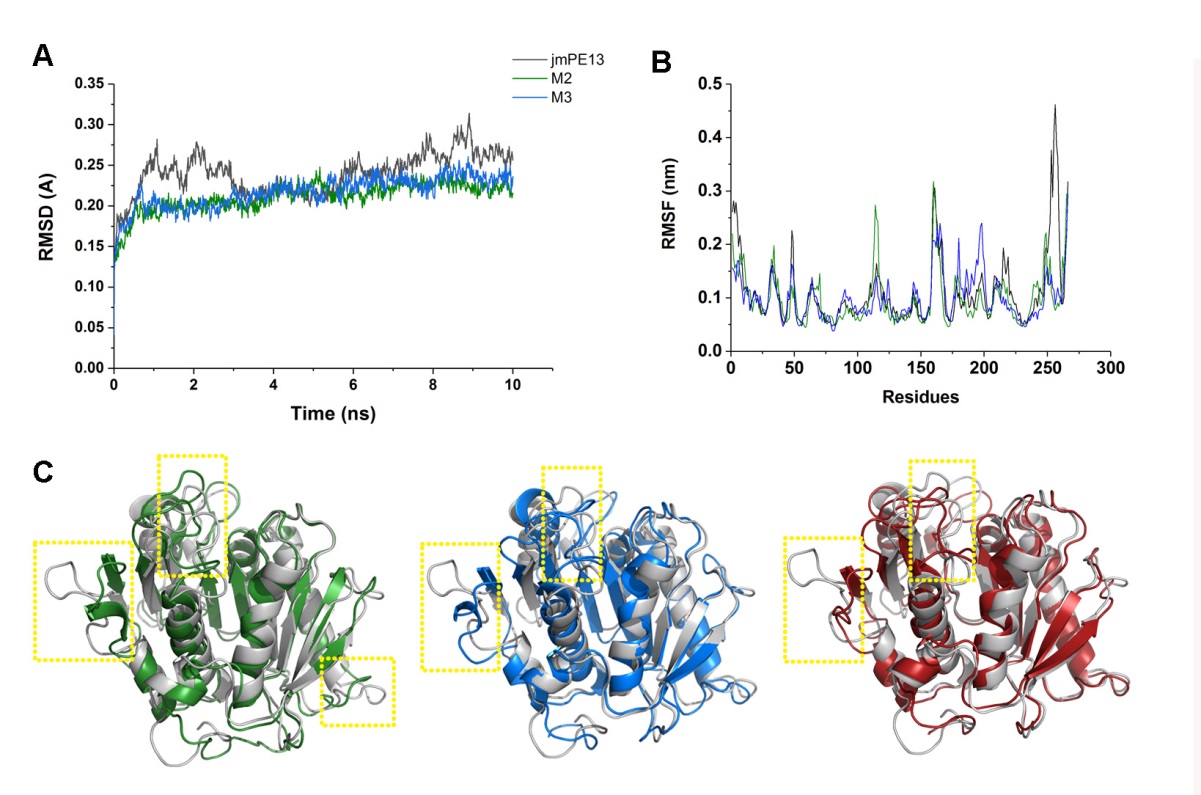


**Supplementary Figure 5.** MD simulations of jmPE13 and the mutants. (A) RMSD of jmPE13 (black), M2 (green) and M3 (blue) in the 10 ns MD simulations. (B) RMSF of jmPE13 (black) and , M2 (green) and M3 (blue). (C) Superposition of the average model structures of jmPE13 and the mutants. grey, jmPE13; green, M2; blue, M3; red, M4. The dashed boxes indicate areas with significant differences.

## Supplementary Tables

Supplementary Table 1. The optimized gene sequences of jmPE13 and jmPE14.

| Enzymes | Optimized gene sequences |
| --- | --- |
| jmPE13 | GCGCCGAGCCCGAGCGCGCCGTGCAGCAACTGCACCCGCGGTCCGAACCCGACCGAAGCGAGCCTGAAAGCGAATAGCGGCCCGTTCAGTGTGAGTCGCTTTAGCGTTAGCGGCTTTCTGCGCGGCTTTGGCAGCAGCACCGTGTATTATCCGACCAACACCACCGGCAAAATGGGCGCGATTGCGGTGATTCCGGGCTATCTGAGCTATGAAAGCAGCATTGAATGGTGGGGCCCGCGCCTGGCGAGTCATGGCTTTGTGGTGATGACCATGAACACCAACACCATTTATGATCAGCCGGATAGCCGCGCGAATCAGCTGAGCAGCGCGCTGGATTATCTGATTAGTCAGAGCAACAGCCGCACGAGCCCGCTGTATAACAAAATTGATAGCACCCGCCTGGGCGCCATTGGCTGGAGCATGGGCGGTGGCGGCAGCCTGAAACTGAGCACGCAGCGCAGCATTAACGCGATTATTCCGCAAGCGCCGTATTATGCGGGCTTTAACCGCTTTGATGAAATTAAAACCCCGGCGCTGATTCTGGCGTGCGAAAGCGATGTGGTGGCGCCGGTGGCGATTCATGCGAGCCCGTTTTATAACCGCCTGCCGAACAGCACCCCGAAAGCGTTTCTGGAAATTAACAACGGCAGCCATTTTTGCGCGAACAGCGGCTATCCGGATGAACGCCTGCTGGGCATGTATGGCATTGCGTGGATGAAACGCTTTATTGATTTTGATACCCGCTATAGTCAGTTTCTGTGCGGCCCGAACCATACGGCGGATTATAGCATTAGCGAATATCGTCAGAACTGCCCGTATTAA |
| jmPE14 | GCCCCAAGTGCGCCGTGTAGTAATTGCACCCGCGGTCCGGCGCCGACCGTGGCGAGCCTGAAAGCGAGCAGCGGCCCGTTTAGCACCGCGAAATTTAGCGTGAGCGGCTATCTGCGCGGCTTTGGCAGCAGCACCGTGTATTATCCGACCAACACCACCGGCAAAATGGGTGCGATTGCGGTTATTCCGGGCTATCTGAGCTATGAAAGCAGCATTGAATGGTGGGGCCCGCGCCTGGCGAGTCATGGCTTTGTGGTGATGACCATGAACACCAACACCATTTATGATCAGCCGGATAGCCGCGCGGATCAGCTGAGCAGCGCGCTGGATTATCTGATTAGTCAGAGCAACAGCCGCACGAGCCCGCTGTATAACAAAATTGATAGCACCCGCCTGGGCGCGATTGGCTGGAGCATGGGCGGTGGCGGCAGCCTGAAACTGAGCACGCAGCGCAGCATTAACGCGATTATTCCGCAAGCGCCGTGGTATAGCGGCTATAACACCTTTAATCAGATTACCACCCCGGCGCTGATTCTGGCGTGCGAAAGCGATGTGGTGGCGCCGGTGGCGAGTCATGCGAGCCCGTTTTATAACCGCATTCCGAACAGCACCCCGAAAGCGTTTCTGGAAATTAACAACGGCAGCCATTTTTGCGCGAACAGCGGCTATCCGGATGAAGCGCTGCTGGGCCTGTATGGCATTAGCTGGATGAAACGCTTTATTGATTTTGATACCCGCTATAGTCAGTTTCTGTGCGGCCCGAACCATACCGCGGATTATAGCATTAGCGAATATCGTCAGAACTGCCCGTATTAA |

Supplementary Table 2. Sequence identities of jmPE13 and jmPE14 with the characterized enzymes.

|  |  | jmPE13 | | jmPE14 | |
| --- | --- | --- | --- | --- | --- |
| Accession | Enzyme name | Query Coverage | Percent Identity | Query Coverage | Percent Identity |
| ACC95208 | PET2 | 88% | 60.00% | 89% | 61.89% |
| UUT36763.1 | Ple629 | 87% | 56.23% | 88% | 58.49% |
| UUT36764.1 | Ple628 | 97% | 53.36% | 97% | 55.48% |
| A0A0K8P6T7 | IsPETase | 86% | 52.67% | 87% | 53.82% |
| Q6A0I4 | Thermobifida fusca Cut2 | 87% | 49.24% | 88% | 48.48% |
| E9LVH9 | Thermobifida cellulosilytica Cut2 | 87% | 49.24% | 88% | 47.35% |
| E9LVH7 | Thermobifida alba cut1 | 87% | 48.86% | 88% | 47.73% |
| AEV21261 | LCC | 86% | 46.54% | 87% | 48.08% |
| Q6A0I3 | Thermobifida fusca bta2 | 90% | 48.18% | 92% | 46.21% |
| W0TJ64 | Cut190 | 87% | 46.77% | 88% | 47.15% |
